# Nanostructured Photothermal Materials for Environmental and Catalytic Applications

**DOI:** 10.3390/molecules26247552

**Published:** 2021-12-13

**Authors:** Huige Chen, Run Shi, Tierui Zhang

**Affiliations:** 1 Key Laboratory of Photochemical Conversion and Optoelectronic Materials, Technical Institute of Physics and Chemistry, Chinese Academy of Sciences, Beijing 100190, China; chenhuige20@mails.ucas.ac.cn; 2 Center of Materials Science and Optoelectronics Engineering, University of Chinese Academy of Sciences, Beijing 100049, China

**Keywords:** photothermal materials, seawater evaporation, photothermal catalysis, layered double hydroxide

## Abstract

Solar energy is a green and sustainable clean energy source. Its rational use can alleviate the energy crisis and environmental pollution. Directly converting solar energy into heat energy is the most efficient method among all solar conversion strategies. Recently, various environmental and energy applications based on nanostructured photothermal materials stimulated the re-examination of the interfacial solar energy conversion process. The design of photothermal nanomaterials is demonstrated to be critical to promote the solar-to-heat energy conversion and the following physical and chemical processes. This review introduces the latest photothermal nanomaterials and their nanostructure modulation strategies for environmental (seawater evaporation) and catalytic (C1 conversion) applications. We present the research progress of photothermal seawater evaporation based on two-dimensional and three-dimensional porous materials. Then, we describe the progress of photothermal catalysis based on layered double hydroxide derived nanostructures, hydroxylated indium oxide nanostructures, and metal plasmonic nanostructures. Finally, we present our insights concerning the future development of this field.

## 1. Introduction

With the ever-growing demand for energy over the last century, there have been alarming and looming threats to humanity caused by the massive consumption of non-renewable energy sources, such as the growing energy crisis and severe environmental pollution [1,2,3]. It is compelling for humanity to explore an alternative to fossil energy. Among all renewable energy sources (tidal energy, wind energy, geothermal energy, etc.), solar energy is the most appealing owing to its inherent and unique advantages of abundance, global sustainability, as well as accessible and clean nature [4].

The full utilization of solar energy represents a challenging task. According to the extensive research focused on solar energy, there are mainly three types of solar energy utilization strategies: solar-to-thermal, solar-to-electrical, and solar-to-chemical conversions. Solar-to-thermal conversion in the realms of solar heat collection [2,5,6], photothermal seawater evaporation [7,8], and photothermal catalysis [9,10] has been addressed by considerable published research reports in recent years. Solar-to-electrical conversion is principally utilized in solar cells [11,12] and photoelectrical catalysis [13,14], while solar-to-chemical conversion is mostly studied in the following three types of up-hill reactions: photocatalytic water splitting [15,16], photocatalytic CO_2_ reduction [17,18], and photocatalytic nitrogen fixation [19].

Photothermal nanomaterials could efficiently convert absorbed sunlight into local heat energy on the surface of materials [20]. Compared to conventional photocatalysts working at room temperature, photothermal catalysts typically have a broader solar spectrum and a much higher local reaction temperature [21]. Besides, photothermal catalysis demonstrates the undeniable advantages of low cost and cleanliness with solar energy as a cleaner heat source than the fossil energy-driven thermal catalysis [22]. The above promotes the broad application of photothermal nanomaterials in diversified realms, especially in environmental and catalytic applications [23].

With the emergence of photothermal effects as the strong development potential in environmental and energy catalysis, many efforts have been focused on nanostructured photothermal materials. Up to now, nanomaterials focusing on the application of photothermal effects of plasmonic structures [24], photothermal catalytic hydrogenation reactions [25,26], photothermal catalytic CO_2_ reduction [21,27,28], and photothermal seawater evaporation [29,30] have been separately reviewed. However, these reviews provide only a one-sided overview of photothermal nanomaterials. A comprehensive summary of nanostructured photothermal materials from the general principle of materials design to state-of-the-art environment and catalytic applications has yet to be undertaken.

In this review, we firstly discuss the forms of solar energy utilization, novel nanostructured photothermal materials, and regulation strategies to efficiently expand the utilization of sunlight. Subsequently, the research significance of photothermal seawater evaporation and the current research status of two-dimensional (2D) and three-dimensional (3D) nanomaterials are presented. Thirdly, the recent advance of photothermal catalysis is reviewed from three aspects: layered double hydroxide (LDH) derived nanostructured materials, hydroxylated indium oxide (In_2_O_3__−__x_(OH)_y_) nanostructured materials, and metal plasmonic nanostructured materials. Finally, the conclusion and outlook of this review are proposed.

## 2. Photothermal Conversion

### 2.1. Solar Energy Utilization

Solar energy is produced from the fusion of hydrogen inside the sun [31] and has the following advantages: It is universal, harmless, enormous, and long-lasting. The sun provides the energy needed by living creatures on the earth directly or indirectly [32]. For example, plants convert solar energy into chemical energy and store it in their bodies through photosynthesis. Plants and animals buried in the ground can be transformed into fossil energies (coal, oil, natural gas, etc.) through the evolution of long periods of time [33]. Although the energy radiated by the sun into the earth’s atmosphere is only one 2.2 billionth of its total radiant energy, it is as high as 173,000 TW, which means that the light energy reaching the earth from the sun in one hour could provide the annual global energy consumption [34]. If solar energy can be efficiently converted and utilized, human society would no longer worry about energy and environmental issues [33,35].

Nowadays, three primary forms of solar energy utilization have been frequently studied. Solar-to-electrical conversion demonstrates its application in the field of solar cells and photoelectric catalysis [11,12,13,14]. Solar cells are devices used to directly convert solar energy into electrical energy with the help of the photovoltaic effect [33]. After more than 100 years of development, solar cells have gone through three stages of development. The first generation of solar cells mainly involve monocrystalline silicon and polycrystalline silicon solar cells. The second-generation solar cells are manufactured with various thin-film substrates, mainly CdTe and amorphous silicon thin-film cells [31,32,36]. At present, the third-generation solar cells are in the research and development stage, among which dye-sensitized solar cells, chalcogenide solar cells, and organic solar cells are promising photovoltaic technologies for both outdoor and indoor applications [37,38,39]. With the growing urgent need to develop sustainable energy sources, photoelectric catalysis is used to convert solar energy into chemical energy for energy production, such as water splitting, CO_2_ reduction, and nitrogen fixation [40,41,42].

Solar-to-chemical conversion is the conversion of light energy into chemical energy by simulating the photosynthesis of plants. Since Fujishima and co-workers used light energy to decompose water on TiO_2_ films in 1972 [43], photocatalytic technology has broadened the application areas of photochemical conversion, mainly focusing on up-hill photochemical synthesis [16,17,18,19]. For example, photocatalytic water splitting mainly uses ultraviolet and visible light in the solar spectrum to decompose water into hydrogen and oxygen [16]. Hydrogen has received great attention regarding the high calorific value and environmental friendliness of combustion products. Therefore, hydrogen production from the photolysis of water is considered an ideal strategy for renewable energy production [44]. In the context of the increasing global CO_2_ concentration year by year and the environmental problems caused by the greenhouse effect, great effort has also been put into photocatalytic CO_2_ reduction for the production of C1 (CO, CH_4_, CH_3_OH) and C2 (C_2_H_5_OH) high value-added fuels [17,45,46]. Nitrogen-based compounds have an essential role in the agricultural and chemical industries. The Haber–Bosch process has harsh reaction conditions, requires high temperatures and pressures, consumes a lot of energy, and pollutes the environment [19]. The photocatalytic nitrogen fixation process can reduce N_2_ to NH_3_ under mild conditions, providing a carbon-free path to safer, cleaner, and sustainable NH_3_ production [47].

The third solar energy utilization strategy is solar-to-heat energy conversion and this has been widely investigated in environmental and energy fields, such as photothermal seawater evaporation [48,49,50,51] and photothermal catalysis [10,28,52]. In contrast to the former two solar energy utilization strategies, of which the efficiency is restricted by the large bandgap and severe charge recombination of the materials, solar-to-thermal conversion based on the photothermal effect of black nanomaterials can effectively utilize the energy of visible and infrared light by converting low energy photons into local heating. The strategy is demonstrated to help drive multiple physical and chemical processes on the surface of nanostructured photothermal materials and will be discussed in detail in Section 3 and Section 4.

### 2.2. Photothermal Materials and Regulation Strategies

**Figure 1:**
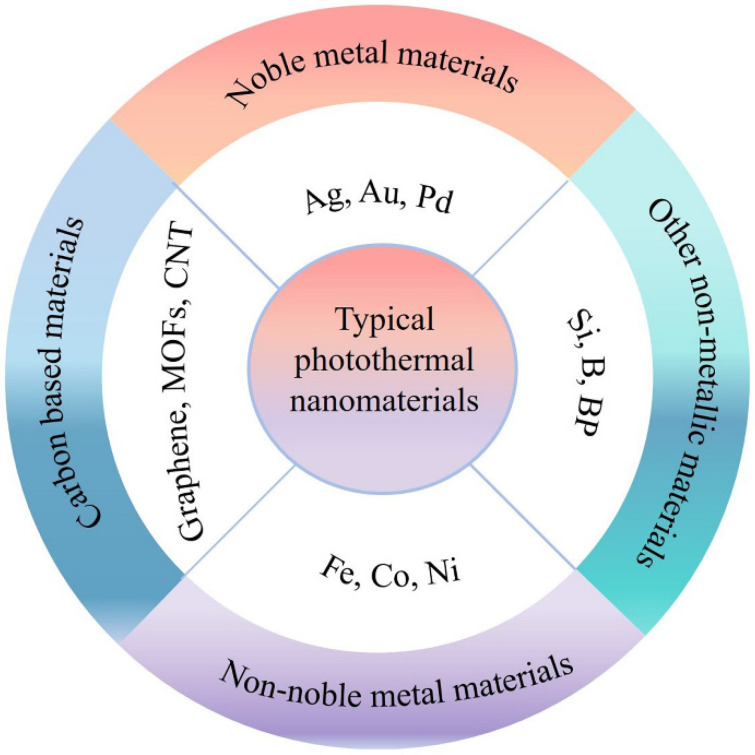
Schematic illustration of typical photothermal nanomaterials.

Theoretically, the potential photothermal materials should have the following characteristics: (i) broad spectrum absorption, (ii) high photothermal conversion efficiency, (iii) processability and physicochemical stability, and (iv) low cost. In practical applications, exploring such ideal photothermal materials remains a very challenging issue. Even so, persistent efforts have been devoted to exploring suitable nanomaterials in favor of photothermal conversion, which can be mainly summarized as noble metal materials [53,54,55,56], transition metal materials [9,57,58], carbon materials [59,60], and other non-metallic materials [61,62,63], as shown in Figure 1. Moreover, corresponding modulation strategies have been investigated for different classes of photothermal materials to further boost photothermal efficiency.

#### 2.2.1. Metallic Materials

Noble metal materials: The local surface plasmon resonance (LSPR) effect of noble metal nanomaterials (Au [53], Ag [54], Pd [55,64]) has developed them into star materials in photothermal effect researches. The LSPR wavelength of metallic materials can be tuned from ultraviolet (UV) to visible (Vis) and near-infrared (NIR) region, which enables the broad spectral absorption of sunlight and its effective conversion to thermal energy by nonradiative damping [48,65]. There is a tremendous challenge for scientists to seek effective strategies to produce upgraded LSPR light absorption characteristics at specific wavelengths [66]. The first strategy is to tune the absorption wavelength region by adjusting the shapes and sizes of noble metal nanostructures. For example, commonly seen Pd nanostructures (nanocage, nanoplate) only show LSPR peaks in the UV-Vis region. For the first time, Zheng et al. synthesized Pd nanosheets with a thickness of less than ten atomic layers by fine-tuning the Pd nanostructure. They found that the ultrathin structure enabled it to have LSPR absorption in the NIR region and convert the absorbed light into heat, which was the main reason for the superior catalytic performance exhibited by Pd nanosheets compared with commercial palladium black [55]. The plasmonic coupling effect of forming hot-spot regions between two metal nanoparticles is the second way to adjust the light absorption property of noble metal nanostructures. For example, the plasmonic coupling effect of Pt and Au on Pt-Au/SiO_2_ material endowed the catalysts to efficiently utilize UV-Vis light energy, generating more hot electrons, thus reducing the activation energy of CO_2_ molecules [67]. The third strategy is to control the interaction between the metal and the support to modulate the structure of the absorption center. The Pd/ZnO catalyst reported by Hong’s group displayed a weak and broad light absorption with its LSPR redshift to near 570 nm, which might be related to the formation of PdZn alloys due to the strong-metal-support-interaction [64].

Non-noble metal materials: Due to the high cost and low earth content of noble metals, non-precious transition metals have recently gained interest in many catalytic reactions. Among the transition metal family, Fe-based, Co-based, and Ni-based nanostructures with CO_2_ hydrogenation activity, Fischer–Tropsch synthesis (FTs) activity, reverse water gas shift (RWGS) activity, and dry reforming of methane (DRM) activity are breaking star materials in the field of photothermal catalysis. The Fe-based catalysts with RWGS and FTs activities are mostly used in CO_2_ hydrogenation reaction studies, which generate high-value hydrocarbons (C_2+_) [68,69,70]. However, the selectivity of the Fe-based catalysts for C_2+_ products is not very satisfactory. To this end, Zhang’s team used a strategy of combining Fe with Co, which shows high C-C coupling ability in FTs, to synthesize CoFe nanoalloys, achieving high C_2+_ selectivity (35.26%) [57]. Recently, the team developed a low-cost Fe-containing catalyst via the hydrogen reduction of MgFeAl-LDH nanosheets [9]. The as-prepared photothermal catalyst composed of Fe and FeO_x_ showed further enhanced C_2+_ selectivity (52.9%). The heterogeneous structure composed of partially oxidized metallic Fe nanoparticles played an important role in improving the C-C coupling kinetics to suppress methane formation. Co-based catalysts are the preferred catalysts for FTs, with the advantages of (i) abundant reserves, (ii) good resistance to sintering, (iii) high selectivity to long-chain liquid hydrocarbons [71]. In the past years, researchers have devoted themselves to studying Co-based catalysts in generating fuels and olefins from FTs. Doping or modification of Co with nonmetallic elements (C or N) is a strategy to adjust the charge density of Co-based nanomaterials and contribute to enhanced photothermal performances. For example, cobalt nanocrystals modified with C with excellent light olefins (C_2–4_^=^) selectivity were reported by Sun et al. [72]. Compared with Fe-based and Co-based catalysts, Ni-based catalysts have relatively high DRM activity and good stability. However, under light irradiation-induced high-temperature reaction conditions, Ni-based catalysts are prone to carbon accumulation on the surface, which is detrimental to the reaction process. Li’s team designed a novel catalyst system by loading SiO_2_ clusters modified with Ni-temporal nanocrystals on mesoporous SiO_2_ to effectively suppress the carbon build-up during the reaction, thus improving the activity and stability of the catalyst [58].

#### 2.2.2. Metal-Free Materials

Carbon materials: In contrast to metallic materials, carbon materials (graphene materials [73], carbon-based single atom materials [60], metal-organic-framework derivates [74], and carbon nanotube materials [75]) have come to represent one of the ideal photothermal materials due to their high specific surface area, good thermal conductivity, broad spectral absorption, and low cost. The strong light absorption ability and high thermal conductivity of graphene are gaining increasing interest for photothermal applications. Recently, several works have reported that the coupling effect of transition metals with carbon materials can enhance photothermal performance [76,77]. For instance, Pd single atom-loaded nitrogen-doped graphene had been synthesized, achieving excellent acetylene selective hydrogenation performance with high acetylene conversion (99%), ethylene selectivity (93.5%), and good photostability [60]. The modification of the topological structure of carbon materials can also enhance the photothermal conversion efficiency. Liu et al. synthesized black monodisperse mesoporous carbon nanospheres with a specific surface area of about 950 m^2^ g^−^^1^ by carbonization of ZIF-8 organometallic framework [74]. The material exhibited excellent photothermal conversion efficiency (33.0%) and outstanding photostability than commonly used photothermal agents (Au, Bi_2_S_3_, Cu_2−x_S) under 808 nm NIR light irradiation. Besides, the construction of the 3D porous structure of carbon materials can increase the light-harvesting capacity to enhance the solar-to-thermal conversion efficiency, such as 3D porous g-C_3_N_4_/GO aerogel [59].

Other metal-free materials: In addition to carbon-based photothermal materials, other non-metallic materials, such as silicon [63,78], boron [62], and black phosphorus [79], have also attracted a lot of attention in photothermal catalysis. Silicon is an earth-abundant non-metallic element and has been well developed in the industry. Ozin’s team used silicon nanowires (Si NWs) as support to load with non-stoichiometric hydroxylated In_2_O_3−x_(OH)_y_ and ruthenium nanoparticles, respectively, and showed that the presence of Si NWs could significantly improve the light-harvesting ability of the composite catalysts and further enhance the reaction rate [63,78]. Boron has demonstrated unique superiority in solar light absorption and photothermal conversion due to its unique band structure. For example, Ye et al. reported for the first time that the strong absorption of boron catalysts in the UV-Vis and even NIR regions, which contributed to the excellent local photothermal effect to directly reduce CO_2_ to CO and CH_4_ [62]. In addition, black phosphorus (BP) with a graphene-like layered structure possesses a broad spectrum of absorption to NIR and is often combined with other materials in certain ways to achieve enhanced light absorption, which is mainly used in photothermal therapy [61]. Liu’s team reported BP-based nanocomposites as NIR-responsive nano-agent-triggered photothermal materials for high-performance cancer photoimmunotherapy [79].

Generally, both physical evaporation and chemical reaction are temperature-correlated processes, of which the efficiency is highly dependent on the local temperature of the microenvironment. The nanostructured photothermal materials discussed above have been intensively investigated in the fields of seawater evaporation-related environmental applications and clean energy catalysis in recent years, and we summarize the structure–property relationship during the processes in the following sections.

## 3. Photothermal Seawater Evaporation

### 3.1. Research Significance and Status

Freshwater resources face severe shortages as the population grows exponentially and water pollution problems worsen. Currently, nearly one-third of the world’s population lacks access to clean water, and it is expected that two-thirds of the world’s population will be in water stress by 2025 [80]. Water scarcity is pushing humanity to seek an attractive solution. The use of solar energy for photothermal seawater evaporation has turned out to be a promising approach to the shortage of water resources [48]. Photothermal seawater evaporation technology can obtain drinkable water from seawater and wastewater with the advantages of eco-friendliness and high freshwater purity [81]. Photothermal seawater evaporation can convert solar energy into local thermal energy on the material surface, providing the required energy for water evaporation. Moreover, people can ulteriorly promote the water evaporation process by increasing the surface temperature and the gas–liquid interface area through photothermal nanostructure modulation. So far, photothermal water evaporation generally has the following three research directions: (i) desalination of seawater, (ii) purification of wastewater, (iii) photothermal steam sterilization [48]. Seawater desalination, as the name implies, is the process of converting seawater into freshwater, using sustainable and non-polluting solar energy to achieve an energy-saving generation of freshwater. Wastewater purification uses photothermal evaporation technology to treat pollutants (e.g., heavy metal ions, phosphates, nitrates, organic dyes, volatile organic compounds, and even coliform bacteria) and achieve environmental remediation while collecting clean water from wastewater. Steam sterilization is a widely used sterilization method. The high-temperature steam generated by photothermal seawater evaporation can play a key role in killing various bacteria. Importantly, efficient evaporation of seawater is inextricably linked to the excellent performance of photothermal materials. In recent years, progress has been made in designing photothermal conversion materials for efficient seawater evaporation. The structure of nanomaterials with excellent photothermal properties can be divided into 2D layered nanostructures and 3D porous nanostructures. Some recent literature on the 2D and 3D nanostructured materials for photothermal seawater evaporation is shown in Table 1.

### 3.2. Two-Dimensional Nanostructures

**Figure 2:**
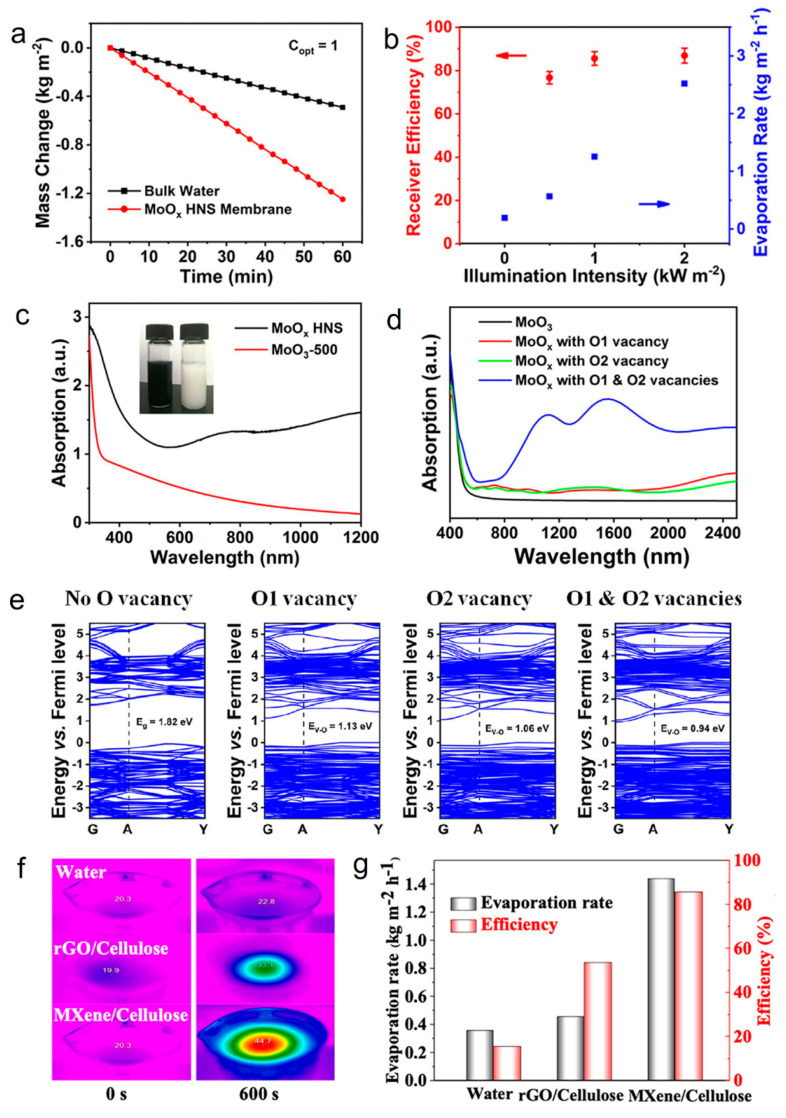
(**a**) Mass change of bulk water and water covered with different MoO_x_ HNS membranes under one sun. (**b**) The evaporation rate and corresponding receiver efficiency of the MoO_x_ HNS membrane under different illumination intensities. (**c**) UV−Vis−NIR absorption spectra of the MoOx HNS and MoO_3_−500 (The MoO_3_ sample was treated via air-oxidation at 500 °C). Inset: optical photos of MoOx HNS (left) and MoO_3_−500 (right) aqueous solutions. (**d**) Simulated optical absorption spectra. (**e**) Simulated band structures of the molybdenum oxides with different concentrations of oxygen vacancies. Reprinted with permission from ref. [49]. Copyright 2019 Wiley-VCH. (**f**) IR thermal images of bulk water, MXene/cellulose, and rGO/cellulose membrane surface. (**g**) Water evaporation rates and solar steam efficiency of bulk water, rGO/cellulose, and MXene/cellulose membranes under the solar illumination of 1 sun. Reprinted with permission from ref. [51]. Copyright 2019 American Chemical Society.

It is well known that the discovery of graphene has brought 2D nanomaterials into the limelight and inspired researchers to explore their properties and applications [102,103]. Recent studies have shown the unique properties of 2D materials, such as ultrathin anisotropy structure, high specific surface area, and tunable optical properties, making them stand out in the field of photothermal seawater evaporation [50]. Up till now, transition metal dichalcogenides (TMDs) [85], transition metal oxides (TMOs) [49], MXenes [51], and other materials in the family of 2D materials have been reported in photothermal seawater evaporation. For example, the strong light absorption and LSPR properties endow TMOs with a high photothermal conversion capacity [50]. Wang et al. reported an atomic-scale thickness of oxygen-defected molybdenum oxides hierarchical nanostructure (MoO_x_ HNS) photothermal nanomaterial for seawater evaporation [49]. The water evaporation rate of the MoO_x_ HNS membrane was 1.255 kg m^−^^2^ h^−^^1^ under one sun irradiation, achieving a high energy conversion efficiency of 85.6% (Figure 2a,b). Furthermore, MoO_x_ HNS with oxygen vacancies and unique flower-like nanoplate structures exhibited broad-spectrum absorption properties (from Vis to NIR light) (Figure 2c), which might be attributed to the electron leap between the valence band, defective energy levels, and conduction band (Figure 2d,e). This study provides a new idea for the future industrial preparation of high-performance and low-cost photothermal seawater evaporation materials.

MXenes is a class of materials consisting of a few atomic layers of transition metal carbides, nitrides, or carbonitrides that has been investigated a lot for photothermal seawater evaporation due to its excellent hydrophilicity, large specific surface area, and electromagnetic wave absorption, which lead to outstanding photothermal conversion properties [104,105]. For instance, Yang’s team synthesized Ti_3_C_2_T_x_/cellulose membrane through the dip-coating method where Ti_3_C_2_T_x_ was coated on cellulose membrane and explored the photothermal seawater evaporation properties of the material [51]. MXenes/cellulose membranes exhibited surprising light absorption efficiency (94%) in the 300–1500 nm spectral range and a higher photothermal conversion efficiency than volumetric water and rGO/cellulose membranes (Figure 2f). It is noteworthy that the MXenes/cellulose membrane achieved an evaporation efficiency of up to 85.6% under one sun irradiation (Figure 2g). In addition, the MXenes/cellulose membranes exhibited good stability under ultrasonic treatment and strong mechanical agitation. In short, the strong light absorption, high photothermal conversion efficiency, good stability, and excellent evaporation efficiency of MXenes/cellulose membranes made it possible to use sunlight for seawater evaporation to obtain clean water in a long-term and sustainable way.

### 3.3. Three-Dimensional Porous Nanostructures

**Figure 3:**
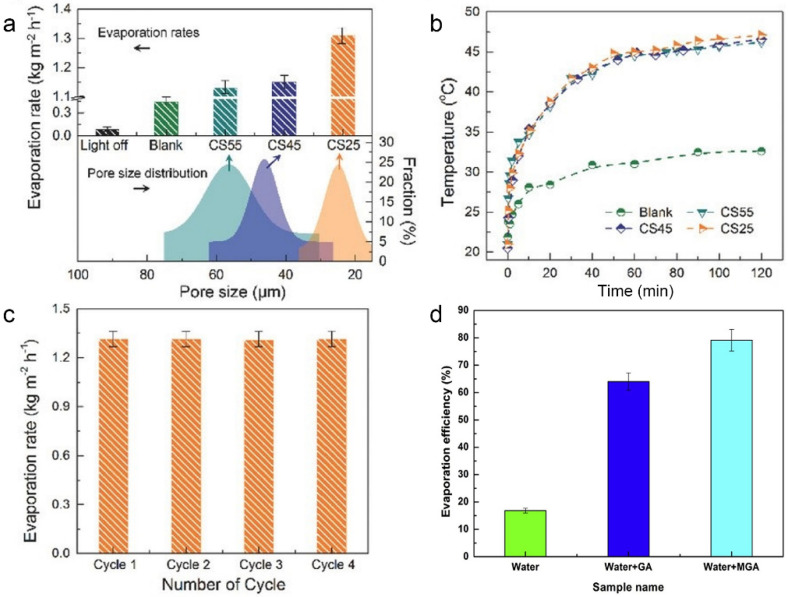
The evaporation rates (**a**) and surface temperature changes (**b**) of different CS at an optical density of 1 kW m^−2^. (**c**) Stability and reusability of CS25 for steam generation. Reprinted with permission from ref. [91]. Copyright 2018 Wiley-VCH. (**d**) The evaporation efficiency of different samples under 1 kW m^−^^2^ solar irradiation. Reprinted with permission from ref. [93]. Copyright 2018 Elsevier.

Although 2D structured nanomaterials show some superiority in photothermal seawater evaporation, as described in Section 3.2 above, such materials still have shortcomings limiting the further improvement of photothermal performance. On the one hand, 2D materials are prone to refraction at the solid–liquid interface due to the Fresnel effect and thus lose light at specific wavelengths [106]. On the other hand, 2D materials have low light reflectivity due to their planar structure, which often leads to poor repeated utilization of light [107]. Hence, 3D porous nanostructures materials such as sponge-like materials [91,92] and aerogels [93] are ideal materials for photothermal seawater evaporation materials due to their pore structure, porosity, and richness of pores, where light can be reflected and scattered multiple times and enhance the light-material interaction, which is an improvement in addressing the shortcomings of 2D materials. Taking carbon sponge (CS) as an example, Ho et al. used a scalable 3D elastic nitrogen-rich porous CS for efficient in situ indirect photothermal evaporation [91]. The CS was floated at the water–air interface so that the photothermal effect of CS heated the interfacial water under light irradiation, and the CS with a minimum pore size of 25 μm (CS25) showed the best photothermal evaporation rate (1.31 kg m^−^^2^ h^−^^1^) after one hour of stable evaporation (Figure 3a) The surface temperature of the CS reached approximately 47 °C (Figure 3b). In addition, the CS exhibited a stable photothermal performance in the cycling test with no significant performance decline (Figure 3c). The broadband optical absorption and the embedded elastic porous pore structure contributed to CS’s ability to avoid excessive heat loss and improve evaporation efficiency. In addition, Wang’s team prepared hydrophilic reduced graphene oxide aerogel (MGA) with photothermal seawater evaporation efficiency of 76.9% under one sun irradiation (Figure 3d) [93]. The hydrophilic 3D network nanostructure of MGA increased the local temperature under irradiation and allowed efficient interfacial water transportation to the photothermal surface, thus contributing to an enhanced evaporation efficiency.

## 4. Photothermal Catalysis

### 4.1. Reaction Types

In addition to the photothermal seawater evaporation described in Section 3, investigations on solar-to-thermal conversion have been extended to the field of photothermal catalysis with the following main applications: decomposition of organic matter [108], hydrogen production [109,110], CO_2_ reduction [111], and CO reduction [52]. Many works have focused on the photothermal catalytic conversion of C1 (CO, CO_2_, and CH_4_) molecules into valuable chemicals and fuels. Specifically, photothermal C1 catalysis is mainly based on the photothermal effect from visible and infrared irradiation to increase the local temperature of the catalyst to overcome the reaction barriers. Photoexcitation under UV irradiation also plays an important role in optimizing product selectivity by modulating the reaction path of reaction intermediates. Various types of reactions have been developed to explore photothermal C1 catalysis. These reactions will be described as follows.

Photothermal catalytic CO_2_ reduction: (i) Methanation of CO_2_ (${\mathrm{CO}}_{2}+4H_{2}\to {\mathrm{CH}}_{4}+2H_{2}O$) [78,112]. CO_2_ reacts with H_2_ to form CH_4_, known as the Sabatier reaction, which is an exothermic reaction. This reaction is promising in energy conversion, especially in the Power to Gas projects. The promising active catalysts for this reaction are usually group VIII metals or metal oxides [28,111]. (ii) RWGS reaction (${\mathrm{CO}}_{2}+H_{2}\to \mathrm{CO}+H_{2}O$) [112]. CO_2_ reacts with H_2_ to form CO and H_2_O. This reaction can effectively convert CO_2_ to CO and is an important way to synthesize high value-added hydrocarbons. In_2_O_3−x_(OH)_y_ and plasmonic metal nanoparticles (Au, Ru, Al, etc.) are often used to study RWGS reactions owing to their unique photothermal and CO_2_/H_2_ activation properties that allow them to exhibit superior catalytic activities compared to other catalysts [28]. (iii) CO_2_ hydrogenation to C_2+_ hydrocarbon reaction. These reactions produce C_2+_ products such as high carbon alkanes (ethane, propane, etc.) by controlling the hydrogenation capacity of CO_2_, which is a promising method for generating high value-added products [28]. (iv) Methanol synthesis (${\mathrm{CO}}_{2}+3H_{2}\to {\mathrm{CH}}_{3}\mathrm{OH}+H_{2}O$) [113]. At present, methanol is generated from syngas at high pressures and temperatures in the industry, which has a large carbon footprint. CO_2_ has been gradually used to replace CO and react with H_2_ for sustainable methanol production. Recently, In_2_O_3−x_(OH)_y_ nanocrystal superstructure [114], Cu/ZnO [115], Na-Co@C [116], Au&Pt@ZIF [117] have been used to study photothermal methanol formation [111]. (v) Dry reforming reaction (${\mathrm{CO}}_{2}+{\mathrm{CH}}_{4}\to 2H_{2}+2\mathrm{CO}$) [118,119]. This reaction converts two major greenhouse gases (CO_2_ and CH_4_) into syngas and subsequently produces clean energy through FTs. Group VIII metals (Rh, Pd, Pt, Ni, Co) have shown good photothermal activity for the DRM reaction [111].

Photothermal catalytic CO conversion: (i) Fischer–Tropsch synthesis. The FTs reaction is a catalytic reaction in which synthesis gas is used as the initial feedstock to produce long-chain alkanes, olefins, and higher alcohols under high temperatures and pressures in the presence of certain catalysts. Over the years, Ni-based, Co-based, Fe-based, and Ru-based catalysts have been extensively studied for FTs [120,121,122,123]. (ii) Water-gas shift (WGS) reaction ($\mathrm{CO}+H_{2}O\to {\mathrm{CO}}_{2}+H_{2}$) [124]. The WGS reaction, which is the reaction of CO and H_2_O to form H_2_ and CO_2_, is gaining attention as a side reaction in the FTs reaction and is widely used in industrial hydrogen production and CO removal as an exothermic reaction [111]. Metal-loaded catalysts (e.g., CuO_x_/Al_2_O_3_) are mostly used to study the WGS reaction [125].

### 4.2. LDH Topological Transformation Nanostructures

LDH is composed of positively charged laminates, interlayer anions, and water molecules, showing a similar structure to hydromagnesite Mg(OH)_2_, and the main laminate is formed by the shared prism of NO_6_ octahedra [126]. The general formula of LDH material is [M_1−x_^2+^M_x_^3+^(OH)_2_]^q+^A_x/n_^n−^·γH_2_O, where M^2+^ and M^3+^ represent divalent and trivalent cations, respectively, and A is the interlayer anion. In general, the common divalent cations forming LDH are Mg^2+^, Ni^2+^, Zn^2+^, Co^2+^, Fe^2+^, Mn^2+^, Ca^2+^, and Cu^2+^, and common trivalent cations are Cr^3+^, Fe^3+^ and Al^3+^ [127]. The intercalated anions between the LDH layers can be chosen from I^−^, F^−^, Cl^−^, Br^−^, OH^−^, CO_3_^2−^, SO_4_^2−^, and PO_4_^3−^ [128].

The outstanding features of variable chemical composition and tunable morphology mean LDH often serve as an effective precursor for highly dispersed metal-loaded catalysts [129]. In recent years, LDH-based photothermal catalysis is mainly focused on CO and CO_2_ hydrogenation [52,121]. Recent literature on the LDH-derived materials for photothermal C1 conversion is summarized in Table 2. For example, Zhang et al. obtained a series of Co-based catalysts with different phase compositions by the thermal reduction of ZnCoAl-LDH with H_2_ at different temperatures (from 300 to 700 °C) [121] Among them, the Co_3_O_4_/Co nanocatalysts obtained by the reduction at 450 °C achieved up to 36% selectivity of C_2–4_^=^ olefins under the light condition. The structural characterization confirmed that the interfacial structure composed of nano-scale Co_3_O_4_ and monomeric Co was the active phase of the reaction. Further, theoretical calculations showed that the Co_3_O_4_/Co interfacial structure weakened the over-hydrogenation ability of metallic Co, thus increasing the selectivity of low-carbon olefins.

**Figure 4:**
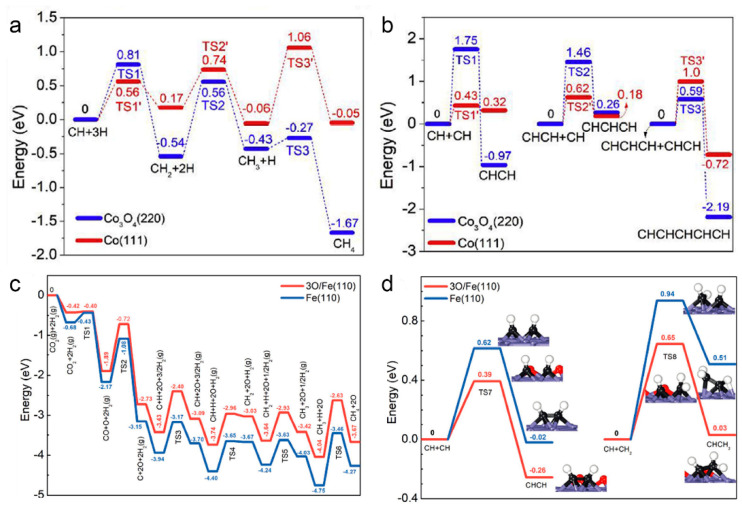
The potential energy profile of (**a**) CH_4_ formation and (**b**) C−C coupling on Co_3_O_4_ (220) and Co (111). Reprinted with permission from ref. [52]. Copyright 2019 Elsevier. The potential-energy profiled of the possible pathways for CO_2_ conversion on 3O/Fe (110) and Fe (110). (**c**) CH_4_ formation and (**d**) C−C coupling reactions. The insets in (**d**) show the configurations of the transition and final states of C−C coupling. The blue, black, red, and white spheres represent Fe, C, O, and H atoms, respectively. Reprinted with permission from ref. [9]. Copyright 2021 Wiley-VCH.

In a subsequent study, Zhang et al. obtained a series of Co-based catalysts with different phase compositions by reducing CoAl-LDH nanosheets in a hydrogen atmosphere under different temperatures (Co_3_O_4_ for temperature below 600 °C and Co(0) for temperature above 600 °C) [52]. Among them, the Co-700 catalysts obtained by reduction at 700 °C achieved up to 65% C_2+_ selectivity (~36.3% C_2–4_ and ~28.7% C_5+_) under UV-Vis irradiation. Density functional theory (DFT) calculations confirmed that the high selectivity for high hydrocarbons was due to the formation of metallic Co nanoparticles that enhance the C-C coupling ability of LDH-derived catalysts (Figure 4a,b). In addition to FTs, photothermal CO_2_ hydrogenation based on LDH nanostructures has also been investigated. Ye et al. reported the photothermal CO_2_ hydrogenation to CH_4_ based on Ru-loaded ultrathin MgAl-LDH in a gas-flowing reactor, achieved a high CH_4_ production rate of 277 μmol g^−^^1^ h^−^^1^ owing to the abundant surface OH groups of LDH to facilitate the chemosorption and activation of CO_2_ molecules [130]. Besides, it was found that the Fe-based nanocatalysts composed of Fe and FeO_x_ formed by reducing precursors at 500 °C had high C_2+_ selectivity (52.9%) for CO_2_ hydrogenation under UV-Vis light irradiation [9]. The results showed that Fe(0) and FeO_x_ were the active phases of Fe-500 catalysts and that the Fe(0)/FeO_x_ ratio was optimal at this point, with excess Fe(0) leading to high CH_4_ selectivity. The significant role of FeO_x_ in modulating the electronic structure of metallic Fe nanoparticles inhibited CH_2_ and CH_3_ over-hydrogenation on Fe(0) nanoparticles, thereby enhancing the C-C coupling reaction (Figure 4c,d). In conclusion, the heterogeneous structure consisting of partially oxidized metal Fe nanoparticles was the main factor that improved the photothermal catalytic FTs selectivity towards C_2+_ products.

### 4.3. In_2_O_3−x_(OH)_y_ Nanostructures

In_2_O_3−x_(OH)_y_ has received great attention as a promising photothermal material due to its unique surface, optical, and electronic properties. Its surface is rich in active sites (oxygen vacancies and hydroxide defects), where the hydroxide defects can efficiently adsorb CO_2_ and enhance the ability of CO_2_ capture and activation in RWGS reaction [132].

**Figure 5:**
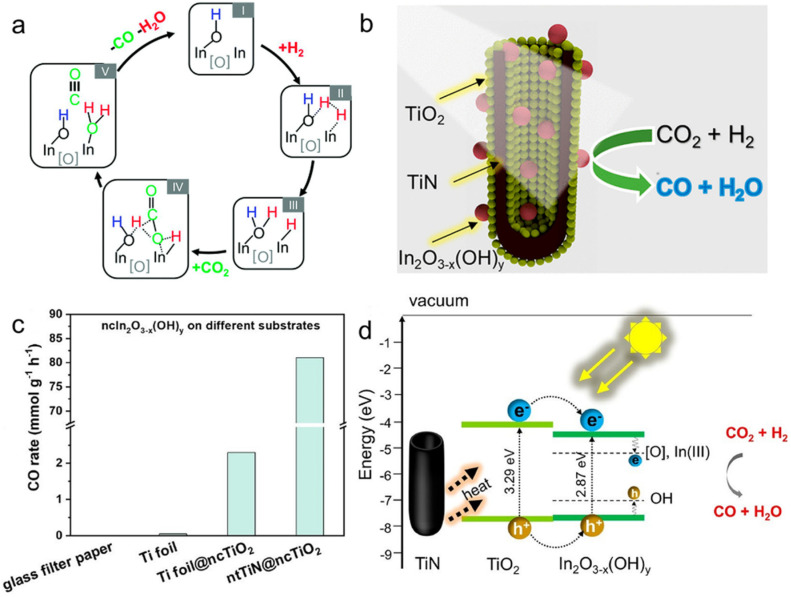
(**a**) Overall proposed mechanism for the RWGS reaction on In_2_O_3__−x_(OH)_y_. Reprinted with permission from ref. [133]. Copyright 2015 Royal Society of Chemistry. (**b**) The schematic diagram of ntTiN@ncTiO_2_@ncIn_2_O_3−x_(OH)_y_. (**c**) nc In_2_O_3__−x_(OH)_y_ supported on different substrates (borosilicate glass microfiber filter paper, Ti foil, Ti foil decorated with ncTiO_2_, and ntTiN decorated with ncTiO_2_). (**d**) Proposed activation mechanism for the photocatalytic reaction. Reprinted with permission from ref. [134]. Copyright 2021 American Chemical Society.

In recent years, many efforts have been made to study RWGS performance over In_2_O_3−x_(OH)_y_ and explore strategies to improve RWGS performance by Ozin’s group [133,134]. Ozin and co-workers investigated for the first time the effect of oxygen vacancies and hydroxide defects on In_2_O_3−x_(OH)_y_ for the RWGS reaction employing the combination of spectroscopic, kinetics, and DFT calculation [133]. They examined the performance of the photothermal RWGS in a flow reactor, and the rate for CO production was 4-fold higher under light irradiation (153 μmol g^−^^1^ h^−^^1^) than under dark conditions (35.7 μmol g^−^^1^ h^−^^1^) at the same temperature (190 °C). The results of this study revealed that the Lewis basic hydroxyl radicals and Lewis acidic indium atoms located near the oxygen vacancies worked synergistically to dissociate H_2_ and activate CO_2_, which subsequently generated CO and H_2_O (Figure 5a). In addition, the increase in RWGS reaction activity could be attributed to the decrease in activation energy due to Lewis alkalinity and Lewis acidity of the excited state enhanced by photoexcited electrons/holes trapped at the frustrated Lewis pairs (FLPs) site. In the following study, in order to reduce the cost of using In_2_O_3−x_(OH)_y_ while ensuring sufficient active site exposure, Ozin’s group also synthesized a series of ternary heterostructured catalysts (ntTiN@ncTiO_2_@ncIn_2_O_3−x_(OH)_y_) via an electrochemical method, where one-dimensional TiN nanotube periodic arrays, TiO_2_ and In_2_O_3−x_(OH)_y_ were sequentially arranged from the inside to the outside (Figure 5b) [134]. The ntTiN@ncTiO_2_@ncIn_2_O_3−x_(OH)_y_ catalyst exhibited an incredible RWGS activity of 81.1 mmol g^−^^1^ h^−^^1^ (Figure 5c). The analysis of the experimental results indicated that the enhancement of catalytic activity was due to the combination of the following three factors: (i) the defective RWGS active sites on the surface of In_2_O_3−x_(OH)_y_ located in the ternary catalyst (ii) the photothermal effect of TiN that provided a photothermal driving force for the reaction, and (iii) the electron transfer between TiO_2_ and In_2_O_3−x_(OH)_y_ that enhanced the CO_2_ hydrogenation ability (Figure 5d).

Recently, In_2_O_3−x_(OH)_y_ has also been used to study the activity of photothermal CO_2_ hydrogenation to methanol [114]. In_2_O_3−x_(OH)_y_ nanocrystal superstructures with 161.21 m^2^ g^−^^1^ specific surface area and 3–7 nm pore size distribution for methanol generation reaction were synthesized by Ozin et al. They explored the effects of different temperatures (200 °C, 250 °C, 300 °C) on the products at atmospheric pressure in a flow reactor. The optimal temperature for the photothermal synthesis of methanol was 250 °C, which reached the highest methanol yield (97.3 μmol g^−^^1^ h^−^^1^) with more than 50% selectivity under light illumination. The main reasons for the high methanol yield were the superstructure of In_2_O_3−x_(OH)_y_ and the unique conformation of the FLPs sites on the surface.

### 4.4. Metal Plasmonic Nanostructures

As discussed in Section 2.2.1, plasmonic nanometals can effectively convert incident UV, visible, and even NIR photons into heat energy through an LSPR mechanism, providing a direct, fast, energy-efficient, and targeted heating method for local reaction sites where catalytic reactions occur [10]. Group VIII nanometals may be the most promising candidate for plasmonic photothermal catalysis.

**Figure 6:**
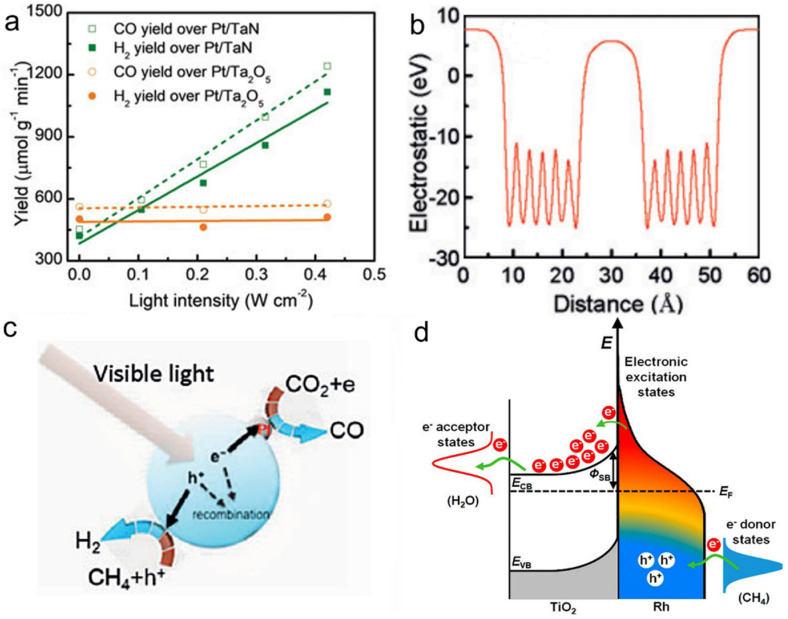
(**a**) Catalytic performance of Pt/TaN and Pt/Ta_2_O_5_ for DRM reaction to produce CO and H_2_. (**b**) The distribution of the electrostatic field over TaN, and (**c**) Proposed mechanism of the DRM reaction over Pt/TaN catalyst under visible light irradiation. Reprinted with permission from ref. [135]. Copyright 2018 Wiley-VCH. (**d**) Schematic of energy transfer from photoexcited hot carriers to adsorbate states and proposed mechanism for SMR reaction on Rh/TiO_2_ under illumination. Reprinted with permission from ref. [138]. Copyright 2018 American Chemical Society.

The light absorption of group VIII plasmonic nanometals covers almost the entire solar spectrum, making them be ideal catalysts for photothermal DRM [28,135,136]. Ye et al. synthesized Pt/TaN catalyst by impregnation method and investigated its catalytic activity with DRM as the reaction [135]. The experimental results showed that the Pt/TaN catalyst had excellent photothermal DRM activity, and the selectivity of the products CO and H_2_ was close to 100% (Figure 6a). In addition, the rate of activity enhancement of Pt/TaN activity was very significant compared with the introduction of Au into Pt/Ta_2_O_5_, which indicated that TaN was an attractive optical body to improve the activity of the loaded catalyst. The reason for the enhanced activity of Pt/TaN catalyst was mainly related to the polar electrostatic field on the surface of TaN. Specifically, as shown in Figure 6b,c, electrons and holes were not easily recombined under the build-in electric field induced by the polarity of TaN. Subsequently, electrons reduced the adsorbed CO_2_ to CO, and holes oxidized CH_4_ for H_2_ evolution, contributing to an outstanding DRM activity. Besides, Ye’s group explored the effect of different particle sizes of nickel loaded on Al_2_O_3_ on the DRM reaction and found that different particle size ranges led to different photothermal activities [136]. Among them, 10 wt.% Ni loading onto Al_2_O_3_ (10 Ni/Al_2_O_3_) catalyst obtained the highest catalytic performance. Under 400–500 nm irradiation, the activity of 10 Ni/Al_2_O_3_ was increased by 1.3 times compared to the catalyst in the dark condition, even at a low light intensity of 0.06 W cm^−^^2^. The LSPR effect was demonstrated to be the main reason for the improved photothermal activity when the size of nickel was less than 17.2 nm, while an interband jumping mechanism on the nickel particles was the dominant reason when the size of a nickel particle was larger than 33.5 nm.

Besides, iron@carbon core-shell nanoparticles with surface-enhanced LSPR effect were obtained owing to the plasmonic coupling between metal core and thin carbon layers [137]. The temperature of the core-shell nanostructure reached 481 °C under irradiation, achieving an RWGS reaction CO yield of 2196 μmol for 120 min. Ye’s team also investigated the steam methane reforming (SMR) activity of Rh [138]. They used an impregnation method to load precious metal Rh nanoparticles on TiO_2_. Under visible light conditions (580 mW cm^−^^2^), the Rh/TiO_2_ catalyst converted CH_4_ to H_2_. With a 50% reduced activation energy compared to catalysis in the absence of light. Experimental characterization and theoretical calculations showed that the hot carriers at the interface between Rh and TiO_2_ could quickly separate, leading to the formation of electron-deficient Rh^δ+^ on their surfaces, thus activating the carbon-hydrogen bond and enabling the further activation of methane at low temperatures (Figure 6d).

## 5. Conclusions and Future Outlook

In this review, we firstly discussed a series of important photothermal nanomaterials (noble metals, transition metals, carbon-based materials, etc.) and proposed the corresponding materials’ modulation strategies to enhance the photothermal performance, and briefly outlined the recent progress of photothermal nanomaterials in the field of photothermal seawater evaporation and photothermal catalysis. Among them, photothermal seawater evaporation was mainly described in terms of the structure of materials (2D structures, 3D structures), while the consideration photothermal catalysis focused on the active site modification over typical photothermal catalytic nanomaterials, summarized the advantages of these materials, and discussed different types of photothermal catalytic reactions. Although research progress has made for photothermal nanomaterials for environmental and energy applications, shortcomings still need to be further addressed.

Firstly, the application of the photothermal effect in non-gas-solid phase catalytic reaction systems (such as liquid-solid phase, gas-solid-liquid triplet phase) has not been thoroughly investigated. Secondly, the application of new photothermal nanomaterials in more energy and environmental processes needs to be further developed. At present, the application of photothermal materials in the environment has only been demonstrated in the field of seawater evaporation. Therefore, future research should focus on combining photothermal catalysis with environmental treatment, such as the photothermal catalytic degradation of pollutants and degradation and conversion of plastics.

In addition to the above mentioned, there are still some shortcomings in the development of photothermal materials: (i) the photothermal mechanism is still unclear, and the role of ultraviolet light in photothermal catalysis is not clear. Photothermal materials should focus on the effect of the coupling between the photothermal effect and the catalytic reaction interface on the photothermal performance; (ii) the test of catalyst surface temperature is not allowed, and the influence mechanism of temperature gradient on photothermal catalytic activity and selectivity at the nanometer scale needs to be studied in depth; (iii) the comparative advantages and application scenarios of photothermal seawater evaporation and photothermal catalysis as compared with traditional water treatment technology and thermochemical industry have yet to be verified; (iv) the measurement standard of photothermal catalytic activity has not been unified; (v) the theoretical simulations on the photothermal effect during interfacial physicochemical processes are still lacking. Given this, developing photothermal materials for environmental and catalytic applications remains ongoing and a lot of research work is still needed.

## Figures and Tables

**Table 1:** Summary of the research on the 2D and 3D nanostructured materials for photothermal seawater evaporation.

Types of Nanostructures	Photothermal Materials	Solar Illumination (kW m^−2^)	Evaporation Rate/Efficiency	Reference
2D nanostructure	Ti_3_C_2_/PVDF membrane	1	84%	[82]
	MoS_2_ nanosheets	0.76	76%	[83]
	BiInSe_3_@CF	1	1.1 kg m^−2^ h^−1^	[84]
	SWNT−MoS_2_ film	5	6.6 kg m^−2^ h^−1^	[85]
	MoOx HNS	1	1.05 kg m^−2^ h^−1^	[49]
	Ti_3_C_2_T_x_/cellulose membrane	1	1.44 kg m^−2^ h^−1^	[51]
	biomimetic Mxene	1	1.33 kg m^−2^ h^−1^	[86]
	2D defective WOx nanosheets	1	78.6%	[87]
	boron nanosheets modified with MoS_2_	1	1.538 kg m^−2^ h^−1^	[88]
	SWNT/AuNR Janus film	5	94%	[89]
	Au/Ti_3_C_2_ membrane	2	2.66 kg m^−2^ h^−1^	[90]
3D nanostructure	cellular nitrogen-enriched CS	1	1.31 kg m^−2^ h^−1^	[91]
	3D graphene foam	1	2.40 kg m^−2^ h^−1^	[92]
	modified graphene aerogel	1	76.9%	[93]
	graphite-modified sponge	1	73.3%	[94]
	RGO−SA−CNT aerogel	1	1.622 kg m^−2^ h^−1^	[95]
	hollow carbon nanotubes aerogels	1	86.8%	[96]
	Co−CNS/MXenes foam	1	93.39%	[97]
	Cu_3_BiS_3_/MXenes	1	1.32 kg m^−2^ h^−1^	[98]
	photo-thermal fiber felt	1	1.48 kg m^−2^ h^−1^	[99]
	cellulose/alginate/carbon black hydrogel	1	1.33 kg m^−2^ h^−1^	[100]
	RGO−SA−cellulose aerogel	1	2.25 kg m^−2^ h^−1^	[101]

**Table 2:** Summary of the research on the LDH-derived materials for photothermal CO/CO_2_ conversion.

Reaction Type	Catalyst	Reaction Temperature (°C)	Rate/Selectivity			Reference
			CO	CH_4_	C_2+_	
CO conversion	Fe−500	230	-	28.6%	60%	[122]
	Co−700	210	-	35%	65%	[52]
	Co−450	195	-	48%	52%	[121]
CO_2_ conversion	CoFe-650	310	4.97%	59.77%	35.26%	[57]
	Ru@FL-LDHs	350	-	277 mmol g^−1^ h^−1^	-	[130]
	Fe−500	275	-	47.1%	52.9%	[9]
	Ni−600	290	-	278.8 mmol·g^−1^ h^−1^	-	[131]
